# Perceptions of deferred blood donors regarding false‐positive screening results for infectious diseases and European blood establishment strategies

**DOI:** 10.1111/vox.70115

**Published:** 2025-09-14

**Authors:** Jenny Erica Beckman, Niubel Díaz Padilla, Afke van der Woud, Peter van den Burg, Vĕra Novotny

**Affiliations:** ^1^ Department of Donor Medicine, Donor Medicine Affairs Sanquin Blood Supply Amsterdam The Netherlands; ^2^ Master's Program in Health Sciences, Specialization in International Public Health Vrije Universiteit Amsterdam Amsterdam The Netherlands; ^3^ Department of Medical Affairs Sanquin Blood Supply Amsterdam The Netherlands

**Keywords:** blood donor, deferral, EBA, FPS, notification letter, psychological distress

## Abstract

**Background and Objectives:**

This study explores the perceptions of deferred blood donors in the Netherlands regarding the information provided upon receiving false‐positive screening (FPS) deferral letters for infectious diseases. To ensure blood supply safety, rigorous screening for infectious agents is implemented by blood establishments. However, FPS results can create challenges after donor notification, leading to psychological distress.

**Materials and Methods:**

The study purposively selected whole‐blood donors who had received deferral letters due to two FPS results between April and October 2023. Semi‐structured interviews, guided by the Health Belief Model (HBM), examined health behaviours through seven constructs. Additionally, a survey of European Blood Alliance (EBA) members was conducted to gather insights on FPS management practices. Interview transcripts were coded and modelled to illustrate relationships between the themes.

**Results:**

Ten in‐depth interviews were held, revealing varied responses: some donors felt reassured by safety protocols, while the majority experienced psychological distress. Key themes included emotional reactions, contact methods, follow‐up communication, engagement with Sanquin Blood Supply and altruistic motivations. The EBA survey shows varied FPS notification practices, rejection protocols, limited donor reaction studies and diverse support measures.

**Conclusion:**

Improving communication clarity, providing follow‐up procedures and adopting best practices from EBA members can enhance donor experience. These efforts are essential for refining the deferral process and improving donor perceptions, ultimately benefiting the donor and the blood supply system.

**Highlights**

- Blood donors who are deferred based on false‐positive screening (FPS) results experience psychological distress, including disappointment, confusion, upset and disapproval.
- Insights from our research, supported by survey findings and the literature, suggest that Sanquin can reduce donor distress and improve understanding by informing donors after the first FPS result using both letters and telephone calls for a more personalized approach.
- The 0.07% of donors who receive two FPS results may benefit from structured follow‐up to ensure adequate information and support.

## INTRODUCTION

Maintaining donor retention is essential for ensuring a sustainable blood supply. However, repeated false‐positive screening (FPS) results can undermine donor trust and discourage donations, posing a challenge for blood establishments.

In the Netherlands, blood donation safety is ensured through a rigorous process [1, 2, 3] involving medical assessment followed by laboratory screening for infectious agents [4, 5]. In case of positive screening with negative confirmatory tests in two donations, donors are notified and temporarily deferred for 2 years [6]. In 2023, the prevalence of two FPS results at our centre was 0.07% [7].

Studies in the United States highlight dissatisfaction among deferral letters, with 81% of donors reporting confusion and seeking clarification from healthcare providers rather than blood centres. Emotional upset was reported by 75% initially, which decreased over time, although 36% still felt upset months later [8]. Similarly, over 80% of Swedish donors expressed concern about insufficient information in FPS deferral letters, despite the dual notification system of letters and phone calls [9].

Canadian donors (86%) also experienced psychological distress upon receiving their FPS result [10], often due to misunderstanding and insufficient information, reducing donor return [11, 12]. Additionally, donors found FPS letters harder to understand than positive results, although both groups experienced similar emotional upset [8]. Miscommunication often leads donors to mistakenly believe they have an infection, as many struggle to understand their deferral reasons [13, 14, 15].

**FIGURE 1:**
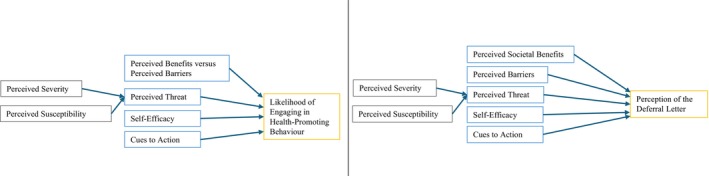
The Health Belief Model (left) and the Revised Health Belief Model (right).

This qualitative study aims to analyse the perceptions of Dutch deferred blood donors regarding FPS result communication and compares these with European blood establishment strategies. Interview and survey topics were based on the Health Belief Model (HBM), which outlines key factors influencing health behaviour (Figure 1) [16, 17, 18, 19].

## MATERIALS AND METHODS

### Study design

A qualitative approach captured the in‐depth experiences of temporarily deferred donors in the Netherlands. Interviews focused on their perception of the deferral letter following an FPS result [20]. Additionally, a small‐scale survey analysed FPS management across European blood establishments. The research team included a senior donor physician and another researcher.

### Participants and procedures

Purposive sampling was used to recruit participants who met the following inclusion criterion: whole‐blood (WB) donors with two FPS results for HIV‐1, HIV‐2, hepatitis C virus (HCV), hepatitis B surface antigen or *Treponema pallidum*, along with a deferral letter issued between April and October 2023 (Appendix 1 in Supporting Information) [21]. From 54 initial donors, 31 met the inclusion criterion, and 20 were selected based on proximity. Thirteen responded to the invitation (Appendix 2 in Supporting Information), and 10 completed the interviews. The intention was to focus on donors with FPS deferrals to gain in‐depth, relevant insights.

The study was conducted from March to July 2024 at Sanquin Blood Supply. In‐depth interviews with deferred donors, guided by the HBM and open‐ended questions (Table 1, Appendix 3 in Supporting Information), were conducted in Dutch and audio‐recorded and lasted 30–45 min. Participants completed a demographic form beforehand (Appendix 4 in Supporting Information). Transcriptions were made using the Atlas.ti software, pseudonymized and supplemented with field notes [22]. Data collection concluded after 10 interviews when saturation was reached, as no new themes or insights emerged.

**TABLE 1:** Topics covered in the interview guide.

Time since receiving the letter
Understanding of the deferral letter
Missed information and desired additions
Experience with the current process
Emotional reactions to the letter
Need for support after deferral
Medical follow‐up after letter receipt
Influence on the perception of own health
Personal impact on well‐being
Meaning of temporary exclusion
Trust in the donation process
Motivation to become and remain a blood donor
Intention to return as a blood donor

This study was approved by the Medical Research Ethics Committee under reference number 2024.0178, with a Data Management Plan and non‐Medical Research Involving Human Subjects Act application approved by Sanquin's institutional review board (Appendix 5 in Supporting Information).

### Analysis

Thematic analysis was conducted using Braun and Clarke's six‐phase framework, guided by the HBM [23]. Recordings were manually transcribed, proofread and analysed with a deductive approach to align codes with HBM constructs [22]. Themes were refined iteratively, and visual tools illustrated the code connections (Appendix 6 in Supporting Information). Inter‐coder reliability was ensured through team discussions.

### EBA survey and analysis

A small‐scale survey was conducted among European Blood Alliance (EBA) members to explore practices related to FPS results, such as notification methods, donor reactions and support services (Appendix 7 in Supporting Information). After pilot testing with staff, it was distributed digitally, and results were descriptively analysed to compare FPS management practices [24, 25].

## RESULTS

Donor perceptions from interviews are presented first, followed by EBA survey results. Ten interviews were completed with Dutch donors, with an average duration of 16.56 min. Participants were 60% women, and the mean age was 41 years (Table 2). Most had donated 11–20 times, with a range of 1 to >50. Half had completed secondary vocational education and the rest held higher qualifications.

**TABLE 2:** Demographic and donation characteristics of study participants.

Category	*N* = 10
Sex	
Male	4
Female	6
Age	
18–24	2
25–39	2
40–54	4
55+	2
Donor history: number of donations (including deferral)	
1 (deferred at first attempt)	1
2–3	2
4–10	1
11–20	3
21–49	2
50+	1
Life stage	
University student	1
Working	9
Origin	
Dutch	10
Education	
Secondary vocational education	5
Higher professional education	2
University education	2
Postgraduate education	1

### Perceived societal benefits

Participants remained committed to donating, driven by altruism and moral duty or influenced by family or profession. Most valued patient safety and praised Sanquin's procedures, with trust even increasing in some cases. Deferrals did not change intention, as participants viewed donations as an essential public good.

I just really wanted to help other people with something that didn't cause me any problems. Like, I don't miss my blood, so if someone else can use it. I thought that was a really nice idea. (Female, 21)

### Perceived barriers

Some participants had questions after reading the letter, unsure which infectious disease it referred to, often assuming inflammation.

But that false‐positive is kind of strange because you don't really know what it's about. I'm now assuming that inflammation values were found then and that it was measured again and that it didn't come up then. (Male, 59)

Most participants experienced negative emotions, including disappointment and confusion, due to the sudden deferral and lack of prior notification. A few, however, expressed understanding, reassurance and satisfaction.

But if there is something clearly wrong in your system, then they simply say, you are not allowed to give for two years. So, I am not doing anything wrong, but I am also not allowed to give for two more years. Yeah, I don't quite get it. (Female, 51)

### Perceived severity

The letter barely altered health perceptions by explaining the testing error and stating that no general practitioner visit was needed. Only one participant expressed concern, which was alleviated after consulting a healthcare family member, while another feared future FPS results.

I wasn't angry. I think I was a little bit confused because you hear a little bit of okay, we don't need your blood anymore, or we can't use your blood. So yes, you doubt a little bit of okay, am I okay? (Female, 30)

### Perceived susceptibility

Participants generally understood the letter and felt unconcerned about their disease risk, although some missed key details, causing confusion and concern, especially among those with other health concerns.

I think I called in response to that. Because I'm not sure anymore if it said what disease I was false‐positive for. And I think that's why I called, to find out. (Male, 57)

### Self‐efficacy

Most participants preferred reminders after the deferral period, expressing difficulty remembering the date or breaking routine. They feared this might reduce their return despite their intention to donate again.

But now you think, yeah, if you don't have to anymore for two years, then you're completely out of rhythm and then you're not going to sign up again very quickly, so to speak. So, from that point of view, I think, if you want more donors, I would actually send a signal like, if you want it, you can do it again. (Male, 59)

A minority had marked their calendars and believed return responsibility should lie with donors, promoting self‐management. They still suggested that reminders after 2 years would be helpful.

### Cues to action

Most participants favoured receiving FPS results via letter, valuing its personal touch and effectiveness, while acknowledging that personal contact with everyone is not feasible. However, some preferred phone calls, citing concerns about lost or impersonal letters.

I think a letter is the best option. I can also imagine that you can't call everyone personally and that you might be busy for a long time. And in a letter, you can clearly state all the information and if people have questions, you can always contact them yourself. (Female, 23)

Participants stressed the need for clearer, more detailed explanations about FPS results and deferral status. While letters or phone calls were preferred, some wanted follow‐up phone calls after receiving the letter.

What false‐positive, what was it tested for? I report every donation, if there is something that is not good, let me know. Then you put that check mark at the front. And then you suddenly get a letter that there is a false‐positive twice, and that you are not allowed to donate for two years. That you think, huh? (Female, 51)

Participants recommended informing new donors about FPS results and deferrals through letters, website updates and intake interviews. Some suggested addressing FPS results only when relevant, to avoid confusion.

### EBA survey results

Of the 25 EBA members, 18 responded (Table 3, Appendix 8 in Supporting Information) [26]. Notification timing varies: 10 inform donors after the first FPS result, 4 after the second and others at different stages. Fourteen use letters and phone calls; others use urgent convocations, delay contact or do not notify. Sixteen do not study donor reactions; two report mixed responses. Eleven apply temporary or permanent deferral, with differing re‐entry rules. Support includes phone contact from eight, counselling from four and a medical helpline from one. COVID‐19 led one establishment to shift from in‐person contacts to letters and phone calls.

**TABLE 3:** European Blood Alliance survey results.

Question	Answer	Quantity (*N* = 18)
When is the donor informed of an FPS result?	After the first FPS result	10
	After the second FPS result	4
	After the first and second FPS result	1
	After the second or third FPS result	1
	After the third FPS result	2
How is the donor informed?	Letter	1
	No communication	1
	Letter and phone call	14
	1× FPS = next blood centre visit, 2× FPS = phone call or visit	1
	Urgent convocation	1
Has your blood bank researched donor reactions to FPS results?	Yes	2
	No	16
If so, what are the general reactions?	Understanding	2
	Rejected	1
	Psychological distress	2
	Struggling in understanding	2
What happens to the donor after an FPS notification?	Temporary deferred, but will be able to donate again in the future	11
	Temporary deferred after 2× FPS, permanently after the 3× FPS	1
	Depends on the test	3
	Temporarily deferred, re‐entry based on risk assessment	1
	Permanently deferred	1
	No answer	1
Are there support services for donors with FPS results?	No	1
	Contact by phone	8
	Counselling	4
	Contact by phone and counselling	1
	Contact by phone and educational material	1
	Contact by phone and educational material and counselling	1
	Contact by phone and option to contact our office themselves	1
	No answer	1
Additional information	Supplements like vitamins, apple cider vinegar concentrate and whey protein were identified as possible causes	
	Donors with FPS are deferred for 6 months (HCV, HIV, syphilis) or 12 weeks (HBV). After, only control samples are taken. If negative, donation resumes; if positive, permanently deferred but may be contacted if new testing methods emerge	
	Freephone line with nurse/doctor for FPS donors, Monday to Friday. Leads to good understanding and no anxieties	
	More scientific information on immunoassays in healthy populations	
	Donor gets letter with explanation of FP and contact details If the donor wishes, they can call/email to discuss the results	
	Before the pandemic, FP donors got results in person	
	Experienced senior nurse practitioners reassure donors about FPS and provide written explanations	

Abbreviations: FP, false‐positive; FPS, false‐positive screening; HBV, hepatitis b virus; HCV, hepatitis c virus; HIV, human immunodeficiency virus.

## DISCUSSION

Deferral due to FPS results negatively impacts Dutch blood donors emotionally. While the deferral letter maintained altruism, trust and intent to return, many participants reported feelings of confusion [8, 10]. These findings are consistent with earlier research. Kleinman et al. [8] found that FPS notifications were harder to understand than confirmed positive results, causing stronger negative emotions. Delage et al. [10] noted that although re‐entry options maintained positive attitudes, it neither reduced distress nor increased the return rate.

Sanquin Blood Supply, as one of the few organizations actively researching donor reactions, is well positioned to improve communication. There is a need for blood establishments to develop strategies to reduce this emotional impact [9, 27].

Participants expressed irritation at the lack of details in their deferral letter, leading to negative emotions [12, 13]. Compernolle [13] highlight that insufficient details often lead to insecurity and confusion, which may deter future donations. Clear and detailed notification is therefore essential for improving the donor experience and encouraging return [13, 14, 28]. Gemelli et al. [14] and Kiely et al. [28] similarly emphasize that unclear FPS notifications contribute to emotional distress and misunderstanding. While Sanquin Blood Supply strives for transparency, there is concern that explicitly mentioning specific tests such as human immunodeficiency virus (HIV) might lead to incorrect associations with serious conditions, which they seek to avoid.

Sanquin Blood Supply informs donors after a second FPS result, unlike most EBA members who notify after the first. Although this approach aims to reduce unnecessary concern, it leads to confusion and distress among Dutch donors, who feel blindsided by the suddenness. Participants suggested that earlier notification could reduce the emotional impact, by framing it as likely due to random or laboratory errors, making a second result less unexpected.

Support for deferred donors varies across EBA organizations. Most offer phone contact, counselling or educational materials, while in the Netherlands support is available only if donors contact a physician. This limited approach may contribute to the negative emotional responses observed.

Many participants valued the notification letters, aligning with research supporting their use [27]. Whittaker et al. [27] recommended clearer wording, involvement of family physicians and follow‐up phone calls. Gemelli et al. [14] found that deferrals at donation centres caused stronger negative emotions than phone notifications, with anger influencing return intentions more than information quality.

Exploring methods like face‐to‐face interactions or urgent convocations could offer stronger emotional support. Personalized communication, as supported by the HBM, may address individual concerns more effectively [29].

Participants recommended follow‐up communication after deferral, suggesting reminders to support eligibility and return rate [1, 11]. Hillgrove et al. [11] indicate that ongoing communication reduces emotional and practical burdens, reaffirms donor value and supports donation habits. Spekman et al. [1] highlight that personalized invitations and clear messaging about temporary deferral increase donor return. These findings underscore the importance of consistent support to keep donors engaged and improve retention, a focus of Sanquin Blood Supply's research efforts.

Interestingly, few participants contacted Sanquin after receiving their deferral letter despite negative emotions, supporting previous studies [8, 9, 27]. Kleinman et al. [8] found that while most donors had questions, few sought clarifications, recommending clearer notifications and counselling. Tynell et al. [9] noted that even concerned donors rarely pursued additional clarification, pointing to uncertainty about their status and limited use of available resources. Improved follow‐up communication and support is essential to address concerns and provide better guidance after deferral. Whittaker et al. [27] found that only 35% of donors contacted the blood supplier after deferral, while 75% consulted their family physicians, emphasizing the need for improved follow‐up communication. In contrast, none of our participants sought help from healthcare providers.

Participants viewed blood donation as a moral duty and intended to return, consistent with prior research [30, 31]. Clear communication and transparency support this intention by alleviating concerns. Ferguson [30] adds that moral obligation can develop into intrinsic ‘warm‐glow’ motivation, influenced by social norms and community reciprocity. Vahidnia et al. [31] also identified altruism as a key motivator among deferred donors due to FPS results, emphasizing its crucial role in sustaining donation behaviours. While some studies report decreased return rates post deferral, our findings show that many donors intend to return [2, 32, 33, 34]. Whether this intention translates into action remains uncertain, highlighting the importance of effective communication and maintaining altruistic motivation.

This study is the first to explore perceptions of deferred blood donors in the Netherlands using semi‐structured interviews and the HBM [35]. Rigour was ensured through data triangulation, saturation and HBM application. Credibility and reliability were supported by member checking, peer feedback, iterative analysis and piloting. Ethical standards were upheld, and detailed methodology supported transferability [36]. Limitations include a predominance of repeat donors, potential recall and social desirability biases and a narrow focus of the HBM, which may have excluded relevant themes [37, 38]. Additionally, responses may differ for donors deferred early in their donation history. Only two individuals declined for personal reasons, not due to negative feelings towards the blood bank, indicating no evident participation bias related to the deferral process.

Participants recommended improving the deferral process by making communication about FPS results clearer and more detailed to address concerns about sudden rejection and unjustified fear. Clarifying the difference between testing issues and actual infections through revised letters and educational resources is crucial [1]. They advised combining letters and phone calls and notifying donors after the first FPS result to ease distress. To encourage return, participants suggested re‐engaging deferred donors after 2 years via letter, email or phone calls. New donors should be informed about FPS results through letters or phone calls, during intake interviews or only after a second FPS result, to prevent unnecessary distress. A general explainer video on the website could further support understanding and reduce negative reactions.

Future research should explore FPS notification strategies across Europe, include diverse regions and both WB and plasma donors and use a longitudinal approach [39]. Veldhuizen and Van Dongen [39] found that plasma donors show higher intention, confidence and conscientiousness than WB donors. Notably, three participants had been deferred for low ferritin levels and speculated a link to FPS results, a connection that remains unclear and requires further research [40].

In conclusion, while some Dutch donors found Sanquin's deferral letters clear, most experienced confusion and distress, highlighting the need for improved communication. Improving clarity, implementing follow‐up procedures and adopting EBA practices are crucial for refining the deferral process and improving donor perceptions, benefiting both donors and the blood supply system.

## CONFLICT OF INTEREST STATEMENT

The authors declare no conflicts of interest.

## Supporting information


**Data S1.** Supporting information.

## Data Availability

The data that support the findings of this study are available on request from the corresponding author. The data are not publicly available due to privacy or ethical restrictions.
