# Supplementary material for: Perceptions of deferred blood donors regarding false‐positive screening results for infectious diseases and European blood establishment strategies
Source: Vox Sang. 2025 Sep 14;120(12):1216–23. doi: 10.1111/vox.70115 (PMC12695447; doi:10.1111/vox.70115)

Appendix 1: Example Deferral Letter Two FPS Results

Dear Ms. «Prefix»«Last Name»,

Following your donation on «Donation_Date», we would like to inform you of the following.

As you know, we test your blood at every donation using screening tests for infectious diseases. If these tests return negative (i.e., normal), your blood is suitable for transfusion and can be given to patients.

If these tests return positive (i.e., abnormal), your blood cannot be used. In your case, one of the screening tests showed an abnormal result. However, additional blood testing confirmed that there is no presence of an infectious disease. Therefore, there is no need for concern.

Your blood incorrectly tested positive in one of the screening tests; this is known as a false-positive test result. Unfortunately, this occurs regularly, though the exact cause is not known. However, it is important to note that this has no implications for your health. More information on this testing issue can be found in the attached document, False Alarm.

Unfortunately, this false-positive test result has occurred repeatedly in your case. Since it has no impact on your health, we did not inform you earlier, assuming it was a one-time occurrence. However, as the false-positive result has now happened multiple times, preventing your blood from being used, it is no longer feasible for you to continue as a blood donor. Therefore, despite the absence of any medical issues, we regret to inform you that we will no longer be able to invite you for donations. Any scheduled appointments will be canceled.

It is possible that this false-positive result may disappear over time. For this reason, we would like to offer you the opportunity to retest in two years. If no false-positive results occur at that time, we can approve you as a donor again. If you wish to do so, you may contact us at that time.

We hope this letter provides you with sufficient information. As the blood bank's tests have produced a false-positive result, there is no need to visit your general practitioner regarding this matter.

According to our records, you have donated blood or plasma «Number_of_Donations» times. This has made a significant contribution to the treatment of sick patients. On their behalf, we sincerely thank you.

We understand that you may have further questions after reading this letter. Of course, we are happy to assist you. You can reach us at the phone number listed at the top of this letter.

Yours sincerely,

Sanquin Blood Supply

Appendix 2: Invitation Letter, Information Letter, Consent Form


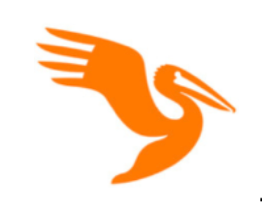
 Instructions for Donors Regarding the Study

Experience After Deregistration as a Blood Donor Due to Testing Issues

Dear Reader,

I hope this letter reaches you well. In the past, you received a letter from us regarding a testing issue involving false-positive results. We are now reaching out to you again, this time to invite you to participate in a study.

We would like to invite you to take part in this research aimed at understanding the experiences of donors who were deregistered due to testing issues involving false-positive results. Our goal is to assess how our communication and services during this process have been perceived and identify areas for improvement if needed.

Your perspective as a donor is invaluable to us. Therefore, we invite you to participate in an engaging interview where we would like to hear about your experience with the rejection process and assess whether our message was clear to you.

Attached, you will find detailed information about the background and purpose of the study. Please take the time to carefully review this information.

Following receipt of this letter, we will contact you by phone within two weeks to discuss whether you are interested in participating in the study. If you are willing, we will schedule an interview and address any questions you may have.

Thank you in advance for your participation!

Kind regards,


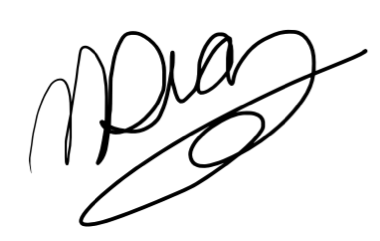


Dr Niubel Díaz Padilla

Senior Donor Physician and Research Coordinator

Sanquin Bloedbank

**Information for Participation in the Study**

**Experience Following Deferral as a Blood Donor Due to Test Issues**

**Introduction**

Dear Reader,

With this information letter, we would like to ask if you would participate in a medical-scientific study. Participation is voluntary.

We are sending you this letter because you were deregistered as a blood donor in the past due to issues with infectious disease screening tests, known as false-positive results. We are interested in learning about your experience with the rejection process.

Your decision to participate or not will have no consequences for your current or future eligibility as a blood donor.

In this letter, you will find information about the purpose of the study, what it involves for you, and what is expected if you decide to participate.

Are you interested?

• Please read this letter carefully.

• Feel free to ask the researcher any questions you may have.

If you would like to participate, please complete the attached consent form.

**1. General information**

The study is being conducted by Sanquin Blood Bank and led by donor physicians from the Donor Medicine department.

**2. Purpose of the study**

Jenny Beckman, a master’s student at Amsterdam UMC (VU), will undertake a five-month internship at the Donor Medicine department. She will investigate the perceptions of donors permanently excluded due to a false-positive result for infectious diseases. The study will involve interviewing donors who were deregistered following two consecutive false-positive results.

The goal is to understand donors’ interpretations of the rejection letter and the psychological impact of receiving it. Additionally, we aim to identify the best way to inform donors about their deregistration. For example:

• Is the letter sufficient?

• Should we use online communication?

• Should we follow up with a phone call?

The study will be supervised by Niubel Díaz Padilla and Vera Novotni.

In this study, we will examine donors’ experiences with being rejected due to false-positive test results in the Netherlands. Specifically, we will explore:

• What donors understand from the rejection letter,

• Their intentions to return as blood donors after being excluded,

• Whether they intend to visit their GP for further information, and

• Their motivations for becoming and remaining blood donors.

As there is no international standard for informing donors about permanent exclusion following false-positive results, we will also compare Sanquin’s practices with strategies used by sister blood banks in Europe.

Sanquin wants to understand donors’ experiences to ensure they do not feel unnecessarily alarmed by the rejection letter or seek unnecessary advice from healthcare providers such as GPs.

**3. What happens if you participate in the study?**

Through interviews, we will ask donors about:

• Their understanding of the rejection letter,

• Their intentions to return as donors,

• Whether they considered visiting their GP for further clarification, and

• Their motivations for becoming blood donors.

We will also collect demographic information such as ethnicity and education level.

**4. What does participation mean for you?**

There are no direct personal benefits from participating in this study. However, please note that participation will take some of your time. The interview will be conducted at a Sanquin donation site and will last approximately 45–60 minutes.

Your participation can contribute to a better understanding of the rejection process and help prevent negative consequences such as stress, anxiety, or unnecessary visits to healthcare providers.

**5. If you do not wish to participate of want to withdraw from the study**

Participation in this study is entirely voluntary. You may withdraw from the study at any time, without providing a reason. However, please inform the researcher promptly if you decide to withdraw. Any data collected up to that point will still be used for the study.

**6. What happens to your data?**

**What does giving consent mean?**

If you participate in the study, you consent to the collection, use, and storage of your data.

**Why do we collect, use, and store your data?**

We collect, use, and store your data to answer the research questions. The results will be published.

**What about audio recordings?**

During the study, we will record audio of the interviews. These recordings will not include identifiable information. After transcription, the recordings will be deleted.

**How is your privacy protected?**

Your data will be anonymized using a code. Directly identifiable information will not be used. The code key will be securely stored at Sanquin, accessible only to the researchers. All data processing and reporting will use this anonymized code. In publications, no one will be able to identify you.

**How long will your data be stored?**

Your data will be stored at Sanquin for 10 years after the publication of the study results.

**Can we contact your GP or specialist?**

If we discover something relevant to your health during the study, the researcher will inform you and may contact your GP or specialist.

**May we request data from the Dutch Central Bureau of Statistics (CBS)?**

If you pass away during the study, we may request information about the cause of death from the CBS if it is relevant to the study.

**Can you withdraw consent for the use of your data?**

You can withdraw your consent at any time, including for future research. However, data already collected for this study may still be used.

**May we contact you for follow-up research?**

We may conduct follow-up studies. On the consent form, you can indicate whether you agree to be contacted for future research.

**Would you like more information about your privacy?**

You can request an electronic copy of your data from the researcher. For more information about your rights, visit Autoriteit Persoonsgegevens.

For questions or complaints about your privacy, contact Sanquin’s Data Protection Officer at privacybloedbank@sanquin.nl, or file a complaint with the Dutch Data Protection Authority.

**7. Questions**

This study has been reviewed by the non-WMO Ethics Committee of Amsterdam UMC. The committee has determined that this research does not fall under the Medical Research Involving Human Subjects Act (WMO).

For questions about the study, please contact Dr. Niubel Díaz Padilla at n.diazpadilla@sanquin.nl.

**8. Complaints**

If you have a complaint, please discuss it with the researchers.

**Thank you for your attention.**

**Contact Details:**

Dr. Niubel Díaz Padilla

Scientific Portfolio Holder, Sanquin

BIG No: 69909030701

Consent Form

To participate, please complete the attached form.

By signing below, I confirm that I have read the information letter and had the opportunity to ask questions. I am satisfied with the answers and had enough time to decide whether to participate.

I understand that participation is voluntary and that I can withdraw at any time, even after providing an interview.

I agree to the use of my research data for the purposes outlined in the information letter.

Name of Participant/Signature: _________________________

Date: _________________________

**Thank you for your cooperation!**

On behalf of the research team,

Dr. Niubel Díaz Padilla

Appendix 3: Interview Guide

Hello, my name is NDP, I am a senior donor physician at Sanquin Blood Bank, and I will be conducting this interview with you. Additionally, JB, my intern from the master program at AUMC, formerly Vrije Universiteit Amsterdam, will be present.

First of all, thank you very much for participating in our research.

Just to clarify, your participation is entirely voluntary, and if at any point you wish to stop the interview, it will be terminated immediately. Furthermore, this conversation will be audio-recorded for research purposes, as described in the information letter.

Do you have any questions? Have you brought and signed the consent form?

Then we can begin.

1. **Motivation**: What motivated you to become a blood donor, and what has kept you motivated to continue donating?
2. **Timing**: How long ago did you receive our letter, and what did you understand from it? (Regarding testing issues)
3. **Experience with the current process:** Could you tell us about your overall experience with the communication process and follow-up after receiving the rejection letter?
   1. **Understanding the rejection letter:** What did you understand from the content of the (rejection) letter (due to testing issues)?
4. **Emotional reactions to the letter:** What feelings did you have upon reading the letter? (e.g., satisfaction, anger, confusion, stress, unease, etc.)
5. **Need for support after rejection:** After being rejected, did you feel the need for support or guidance? If so, in what way?
6. **Medical follow-up after receiving the letter**: Did you consider visiting your general practitioner or other specialists after receiving the letter?
   1. **Impact on perception of personal health:** To what extent did the rejection letter influence your perception of your own health?
   2. **Personal impact on well-being:** How did this experience affect your emotional well-being? Did you use any specific coping strategies?
   3. **Significance of temporary exclusion**: What does it mean to you that you are currently temporarily unable to donate blood?
7. **Missing information and desired additions**: What information did you feel was missing from the rejection letter that you would have found helpful?
8. **Communication of risks:** How would you prefer that risks, such as false-positive results, are communicated to potential donors before they give blood?
9. **Trust in the donation process:** Has receiving the rejection letter affected your trust in the donation process? If so, in what way?
10. **Role of aftercare**: What forms of aftercare or follow-up would you like to see following a rejection due to a false-positive result?
11. **Best practices for informing donors:** What do you think is the best approach to inform donors about exclusion (for two years) after a false-positive result? (e.g., via letter, email, telephone)
    1. **Improvements to the communication process**: How do you think the process of informing donors about temporary exclusion can be improved to foster better understanding and acceptance?
12. **Intention to return as a blood donor:** What is your intention regarding returning as a blood donor after exclusion due to a false-positive result? Has your motivation changed?

Appendix 4: Demographic Form

Name:

Date of Birth:

Gender: What is your gender? Male / Female / Other, …

Ethnicity: What is your ethnic background?

Education level: What is the highest level of education you have completed? Primary education / Secondary education / Secondary vocational education (MBO) / Higher professional education (HBO) / University education (WO) / Postgraduate education (PhD) / Other, …

Profession: What is your occupation?

Residence: Where do you live?

Donation history: Since when have you been a blood donor?

Are you also a stem cell/tissue or organ donor?

Medical history relevant to blood donation:

Experience with healthcare: How would you rate your experience with the healthcare system? Poor / Average / Good

Socio-economic status: How would you describe your current socio-economic status? Low / Average / High / Other, …

Life phase: Which phase of life are you currently in? Secondary school student / University student / Employed / Household tasks (not in paid employment) / Retired / Other, …

Appendix 5: Data Management Plan

**1. Study description and data collection**

The project aims to investigate the impact of false-positive screening (FPS) results on blood donors' experiences in the Netherlands. Despite efforts to alleviate psychological distress in deferred donors, little is known about the specific effects of FPS results on factors as information clarity, communication strategies and emotional response.

The study will involve purposefully recruiting whole blood donors in the Netherlands who have received two positive screening results but a negative confirmatory test (FPS) and have been temporarily deferred. Semi-structured, in-depth interviews will be conducted with these donors to explore their experiences, motivations, and perceptions regarding the deferral letter and FPS result. Additionally, an online survey will be distributed to European Blood Alliance (EBA) organizations to gather information on FPS management practices across Europe.

**2. Planning: the stages of your project**

This study will collect data by doing semi-structured interviews and thematic analysis guided by the Health Belief Model. The software Atlas.ti will help in doing in-depth thematic analysis and manage the coded data. To maintain structure the data, a folder and file naming system will be implemented. For each participant, their name will be pseudonymized and labeled as Participant 1, Participant 2, and so on, up to Participant 10. Each participant will have a separate folder labeled with their pseudonym (e.g., P1, P2). Within each participants’ folder, subfolders will be created for raw data, transcriptions, and analyzed data.

Quality assurance will be integral to maintaining data consistency and accuracy. This includes: a standardized interview guide will be used for all interviews to maintain consistency in data collection, selected interviews may be repeated to verify consistency and data quality, transcriptions will be cross-checked by a second researcher, who will also conduct analysis, and any discrepancies will be discussed and resolved.

Raw data:

- Audio recordings of interviews with donors
- Completed survey responses from EBA organizations
- Researcher notes in an online environment

Processed data:

- Transcribed interviews, coded interview transcripts
- Cleaned and organized survey data into data spreadsheets containing survey responses

Analyzed data:

- Thematic content analysis findings
- Data visualizations in Atlas.ti

Other: study presentations at the university, research publications from Sanquin Blood Supply

**3. Data assets**

The study will collect data from 10 participants, conducting semi-structured interviews with donors temporarily deferred due to FPS results. The data will include audio recordings of interviews stored in MP3 format, transcribed texts saved as DOCX files, and survey responses organized in spreadsheets. These formats have been selected for their compatibility with Atlas.ti for data analysis, and suitability for long-term accessibility and preservation. In total, the collected data is estimated to occupy approximately 1 GB of storage space. This includes around 500 MB for MP3 audio recordings, 200 MB for DOCX transcriptions, and 300 MB for survey data in spreadsheets. Additionally, interviews recorded via Microsoft Teams will be securely stored on that platform. After data collection, data will be analyzed using Atlas.ti. Expected file size: 5-10 GB. Audio recordings of interviews will be saved in MP3 format on a cell phone. This method is chosen for its widespread compatibility and efficient compression, facilitating easy playback and transcription using standard audio software. The recordings are directly transcribed and anonymized. After the study ends, recordings are removed from the audio device. Transcribed texts will be stored in DOCX format, which supports editing, formatting, and sharing among researchers. Qualitative data analysis will utilize Atlas.ti software, storing files, used for coding and thematic analysis. This research does not use existing data regarding perceptions of donors.

**a. Data assets:**
Interviews:

- Audio files: MP3 recordings of interviews on a cell phone.
- Transcribed text files: DOCX files of transcriptions.
- Transcript analysis: Atlas.ti files for coding and analysis.

Survey: completed survey responses: spreadsheets and files with organized survey data.

**b. File formats:**

- Audio files: MP3. Recordings of interviews as part of the research. Each audio file corresponds to a specific interview session may range in length depending on the duration of the session. These files serve as the primary source of data for transcribing and analyzing participants' responses.
- PowerPoint files: PPTX
- Transcribed text files: DOCX
- Atlas files: Atlas.ti. Files generated by qualitative data analysis software for coding and analyzing textual data.
- Online files: DOCX, PPTX, PDF, to write and present the research.

**4. Data (risk) classification:**

- Low risk: survey responses and aggregated data sets.
- Medium risk: transcribed interview texts.
- High risk: personally identifiable information and sensitive data discussed during interviews.

**5. Methods / standards/ protocols for collection and/or analysis**

To address ethical issues and ensure data preservation and sharing are conducted responsibly, we will take several measures. First, all participants will be required to sign a consent form prior to the interview, clearly outlining the purpose and scope of data use, including preservation and sharing. To protect participants' identities, we will pseudonymize all names by assigning each participant a number (e.g., 1, 2, 3) and storing these identifiers in separate folders. Only this code will be used for analysis, guaranteeing that the original data - which contains participant identities - remains inaccessible. Transcripts and audio files will be organized together to maintain structure and prevent unauthorized access.

- Informed consent: obtained from all participants before the interviews by sighing the informed consent form.
- Verbatim transcription: transcription of audio recordings capturing exact words spoken during interviews and focus group discussions.
- Data anonymization: removal or de-identification of personally identifiable information from transcripts and survey data.
- Inter-researcher check: peer review process during thematic content analysis to ensure consistency and accuracy in data analysis.

**6. Storage**

For researchers involved in the study or those who require specific data, access will be managed via Microsoft Teams and Sanquin’s internal servers, where the dataset and related documentation will be securely stored on a secured server using encryption, password protection, and access control. Data sharing will be controlled by permissions set by the project lead, ensuring that only authorized users can access the data.

**a. Where will the data be stored?**

The data collected during the study will initially be stored and managed by the internship placement, Sanquin Blood Supply. They will ensure that the collected data is securely stored on a server using encryption, password protection, and access control measures to safeguard participant confidentiality. Interviewees will be informed in the information letter and consent form they received prior to the interview about how their data will be used and stored. They will also be informed of their rights regarding their data, including how to access it and request changes. If interviewees wish to obtain their anonymized research data, they needed to submit a written request to the researcher.

**b. Draft versions**

- Access to the data of the draft version will be restricted to authorized individuals involved in the research, including researchers, research leaders, and management personnel directly associated with the study
- Access to the data will be protected through security measures to ensure confidentiality and prevent unauthorized access, including encryption of stored data, password protection for digital files and databases, and restricted physical access to any hard copies of data.
- Access to the data will be assigned by the researchers Jenny Beckman and Niubel Diaz Padilla, who oversee the study. Access permissions will be granted based on the specific roles and responsibilities of individuals within the research team.

**c. Final versions**

- Access to the data of the final version will be restricted to authorized individuals involved in the research, including researchers, research leaders, and management personnel directly associated with the study.
- Access to the data will be protected through security measures to ensure confidentiality and prevent unauthorized access, including encryption of stored data, password protection for digital files and databases, and restricted physical access to any hard copies of data.
- Access to the data will be assigned by the researchers overseeing the study, Jenny Beckman and Niubel Diaz Padilla. Access permissions will be granted based on the specific roles and responsibilities of individuals within the research team.

**d. Archiving**

The data collected during the research will be stored for a period of 10 years at Sanquin Blood Supply following the publication of the research results in a scientific journal. During this time, the data will be securely maintained according to established data management and security protocols to prevent loss, unauthorized access, or breaches of confidentiality. The privacy policy of Sanquin Blood Supply describes the procedures for securely deleting data.

The commissioner of the project, Sanquin Blood Supply, will keep the final responsibility over the data protection and storage.

Appendix 6: Visualization of Themes and Codes


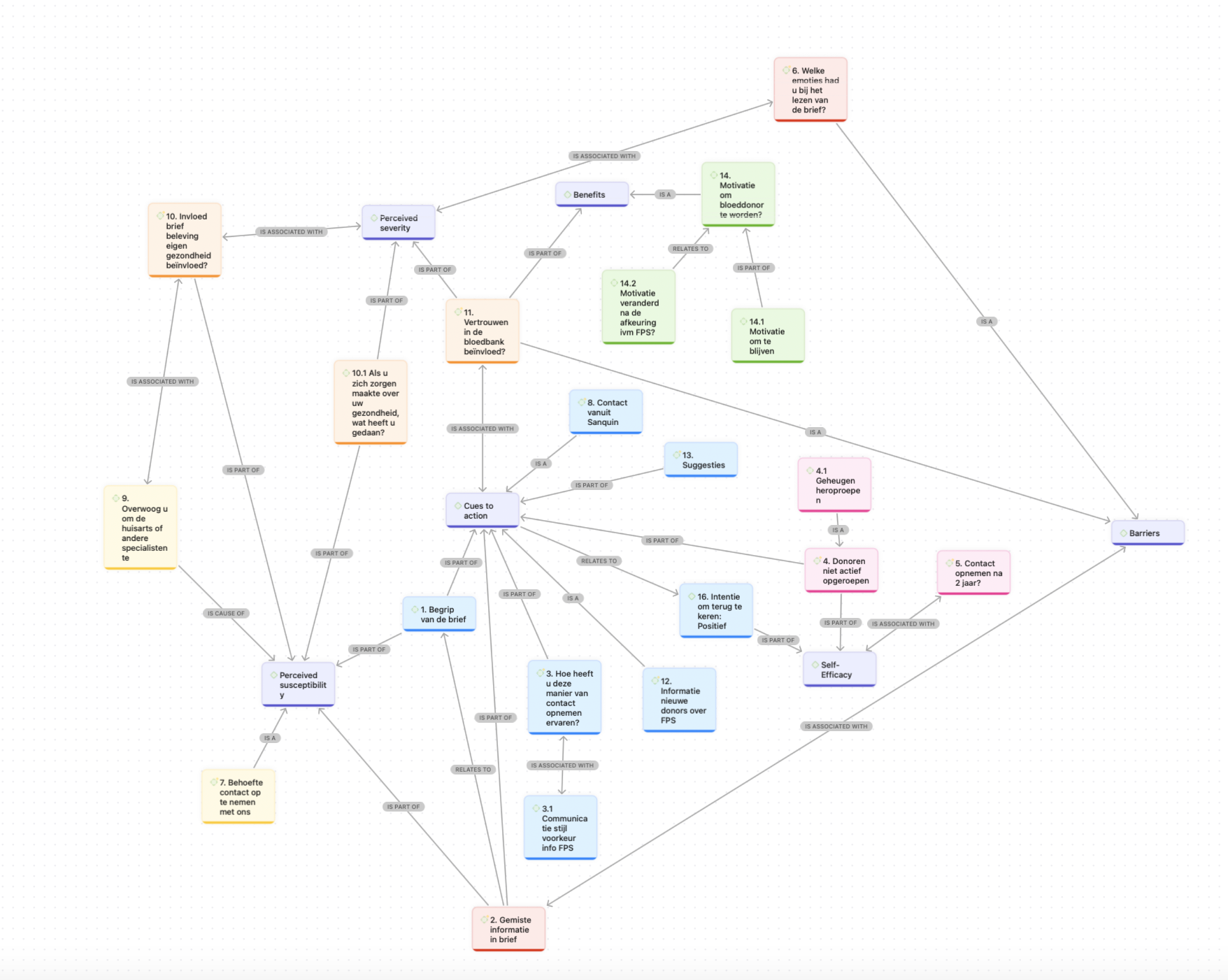


Appendix 7: Blood Bank Survey: False-Positive Screening Results


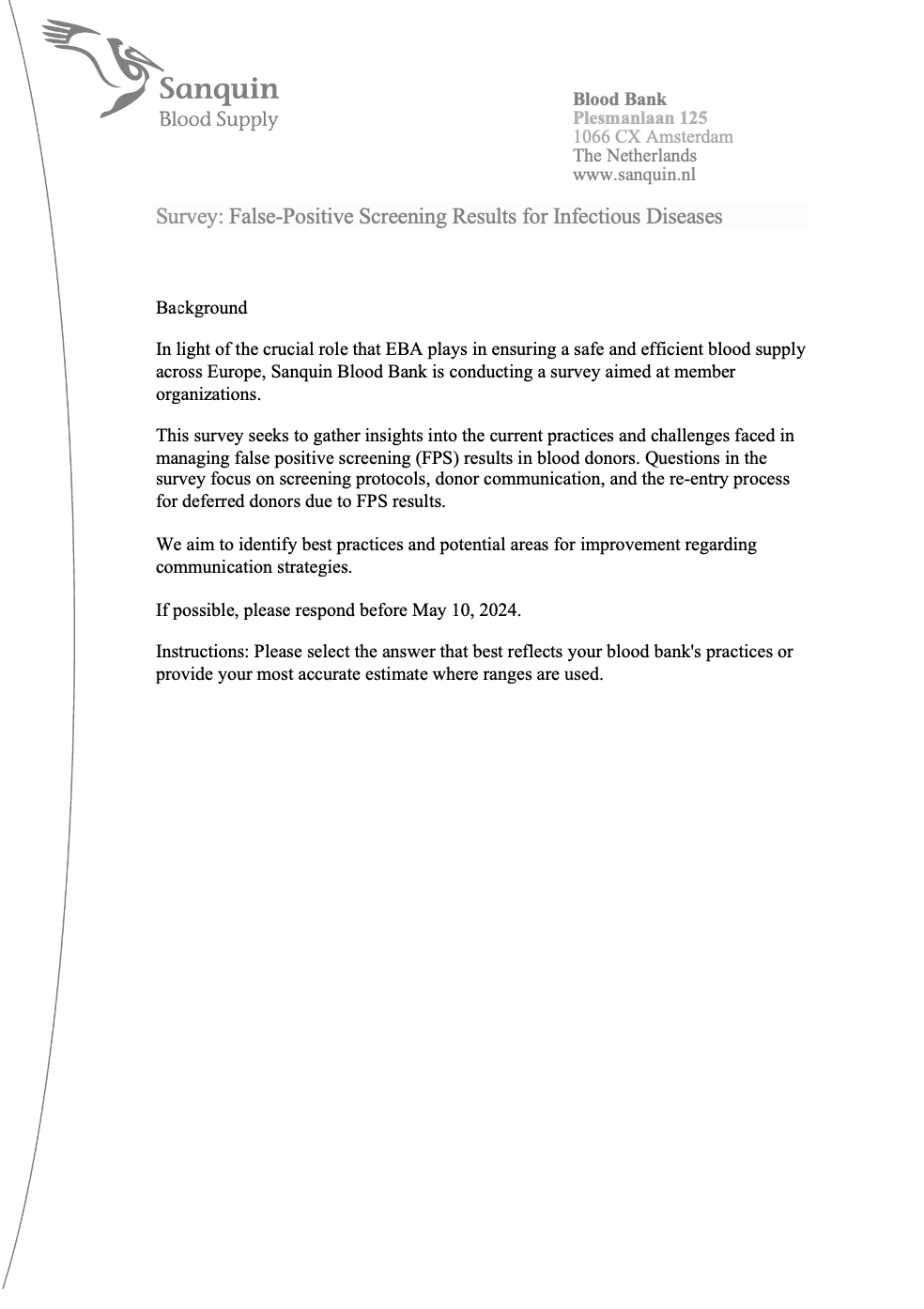


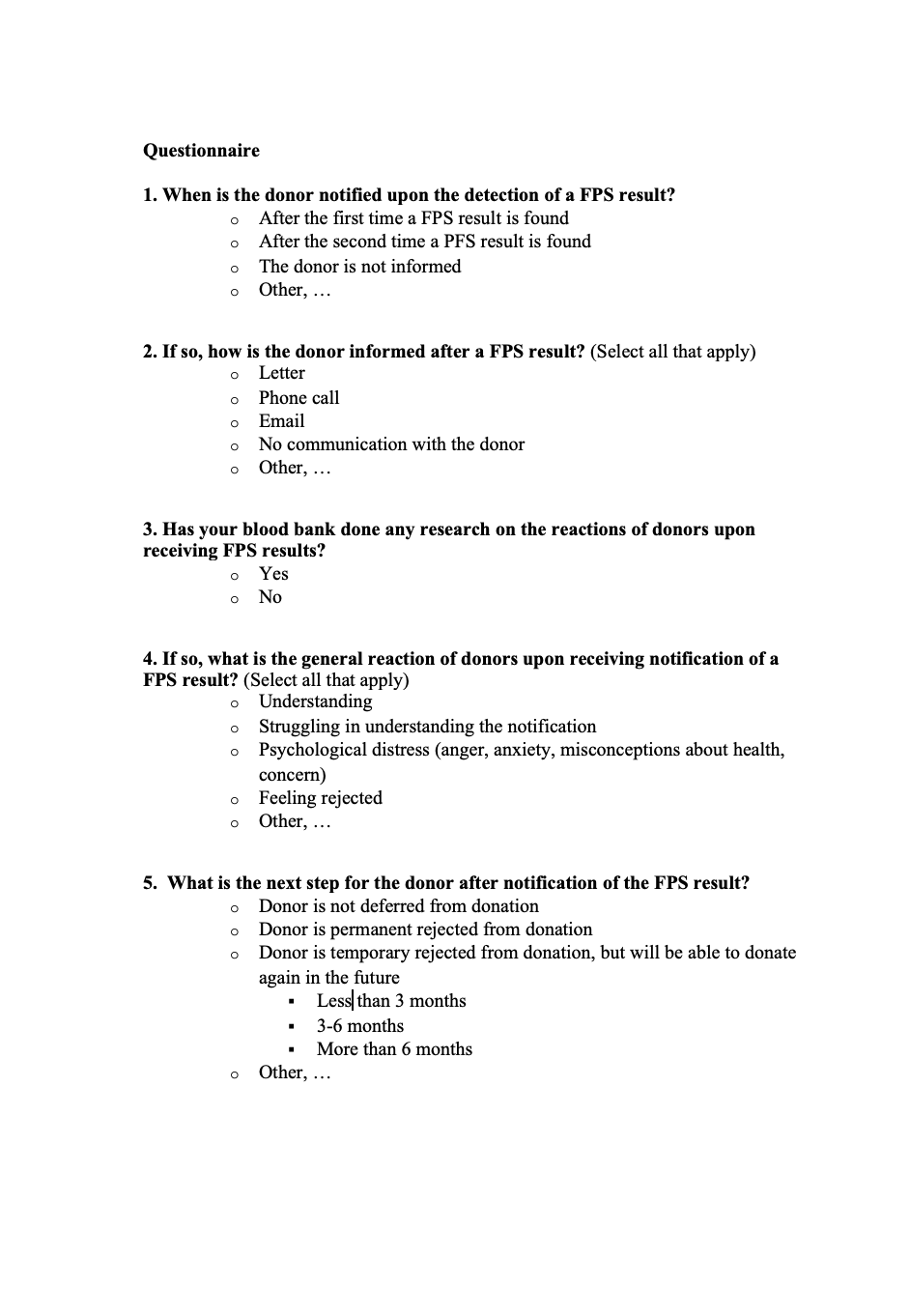


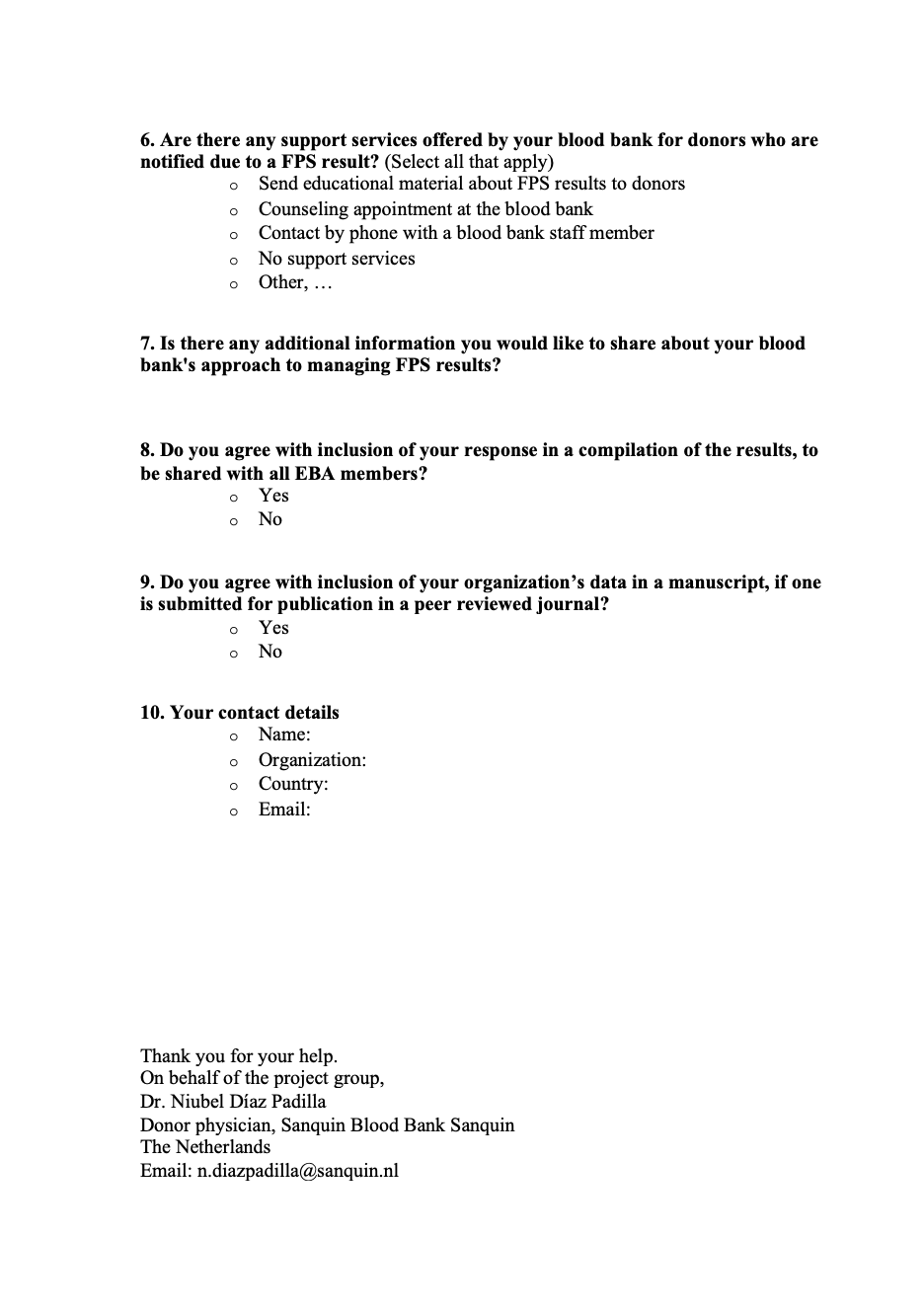


Appendix 8: Table of EBA Survey Results


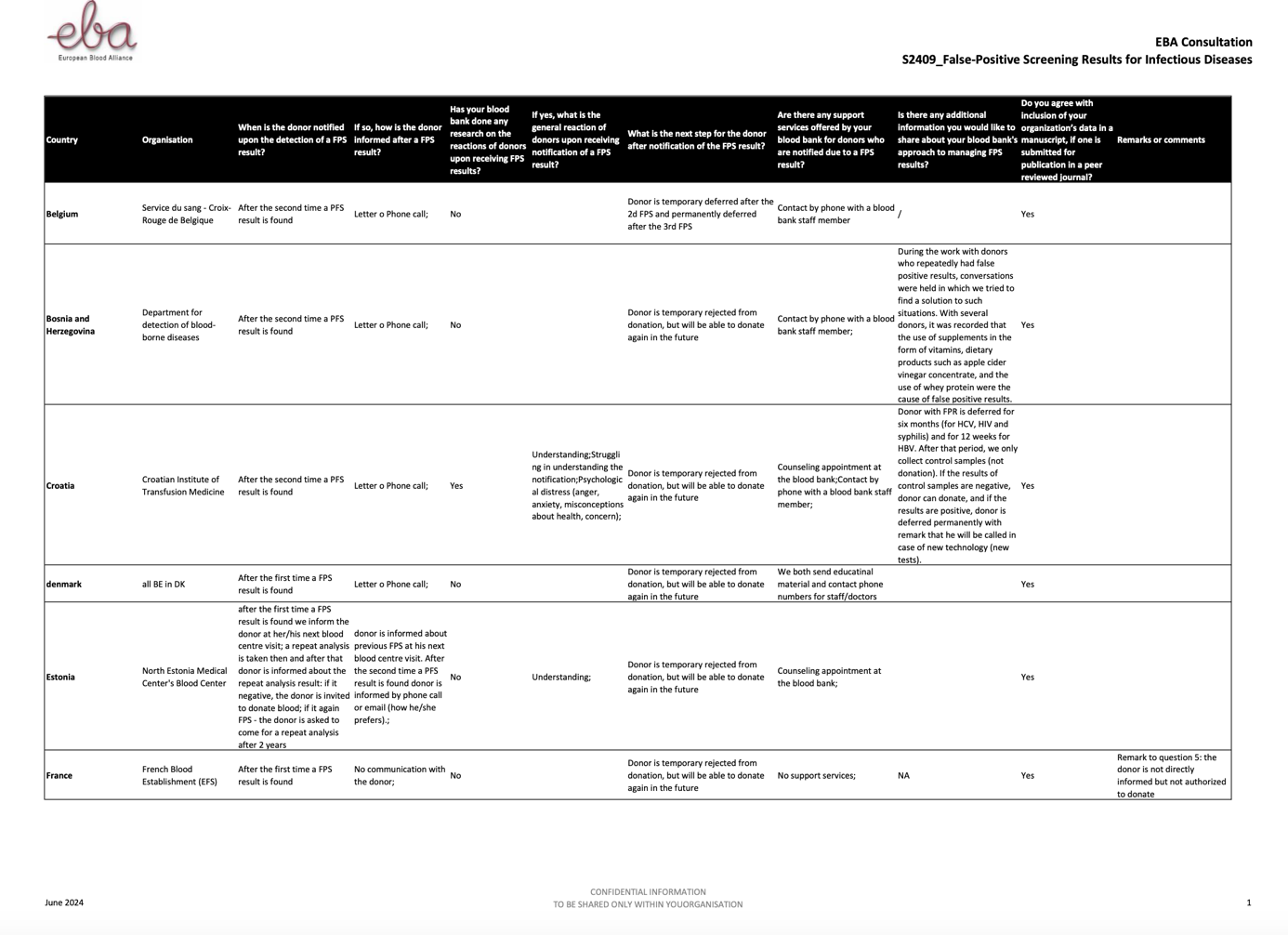

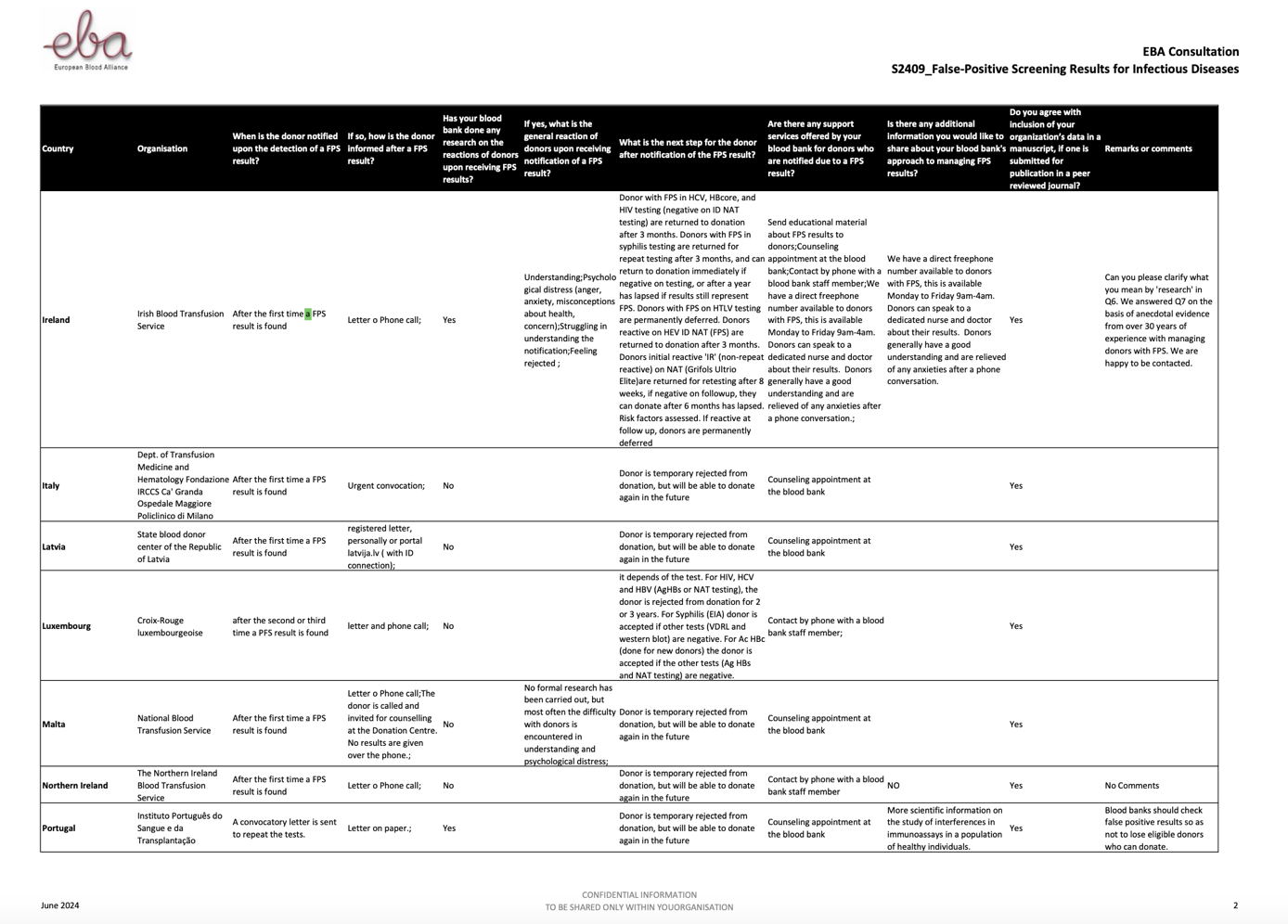


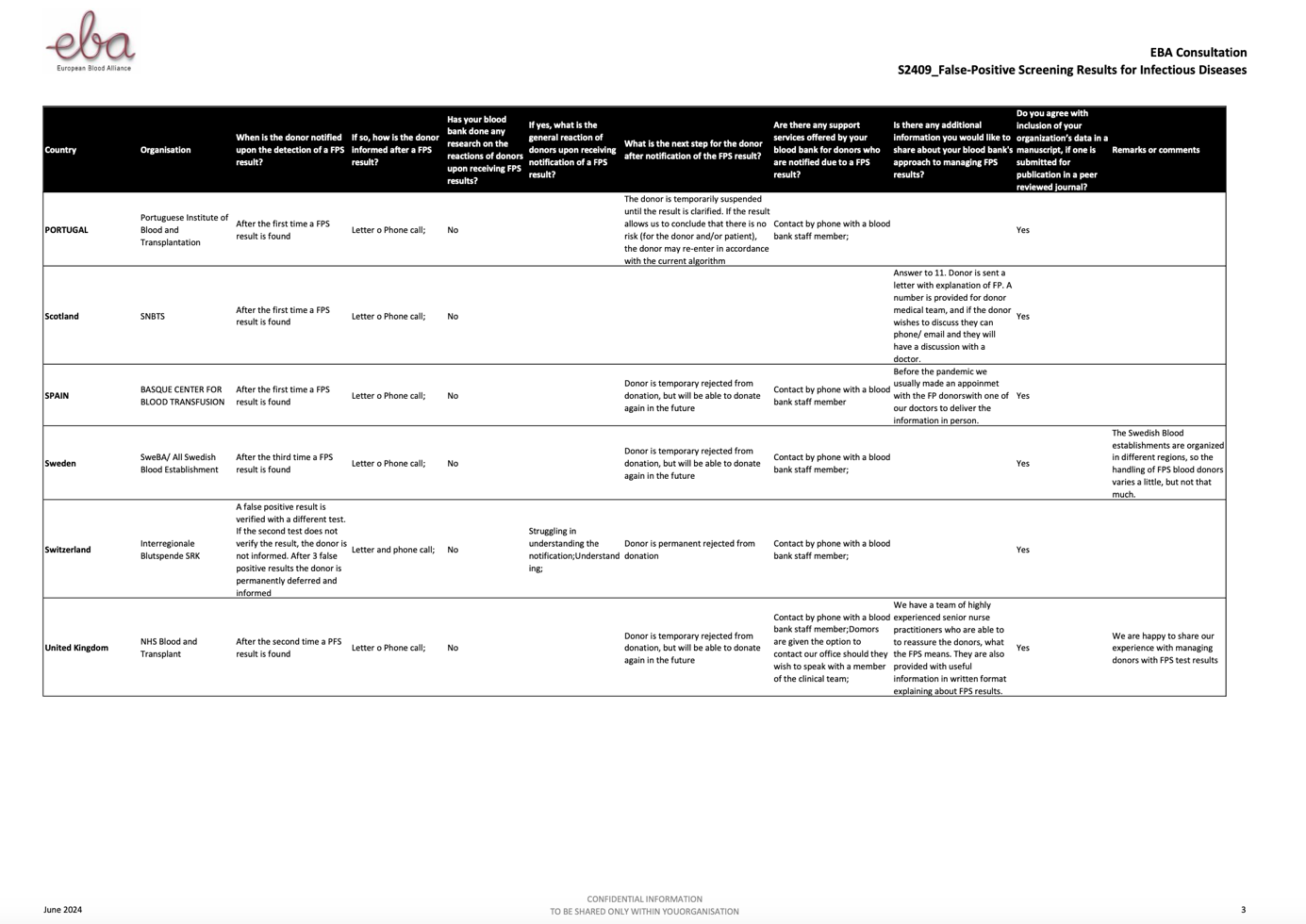

Supplement: Supplementary file 1 — Data S1. Supporting information. [file VOX-120-1216-s001.docx]
